# True Random Number Generation from Bioelectrical and Physical Signals

**DOI:** 10.1155/2018/3579275

**Published:** 2018-07-02

**Authors:** Seda Arslan Tuncer, Turgay Kaya

**Affiliations:** ^1^Department of Software Engineering, Faculty of Engineering, Fırat University, 23119 Elazig, Turkey; ^2^Department of Electrical-Electronics Engineering, Faculty of Engineering, Fırat University, 23119 Elazig, Turkey

## Abstract

It is possible to generate personally identifiable random numbers to be used in some particular applications, such as authentication and key generation. This study presents the true random number generation from bioelectrical signals like EEG, EMG, and EOG and physical signals, such as blood volume pulse, GSR (Galvanic Skin Response), and respiration. The signals used in the random number generation were taken from BNCIHORIZON2020 databases. Random number generation was performed from fifteen different signals (four from EEG, EMG, and EOG and one from respiration, GSR, and blood volume pulse datasets). For this purpose, each signal was first normalized and then sampled. The sampling was achieved by using a nonperiodic and chaotic logistic map. Then, XOR postprocessing was applied to improve the statistical properties of the sampled numbers. NIST SP 800-22 was used to observe the statistical properties of the numbers obtained, the scale index was used to determine the degree of nonperiodicity, and the autocorrelation tests were used to monitor the 0-1 variation of numbers. The numbers produced from bioelectrical and physical signals were successful in all tests. As a result, it has been shown that it is possible to generate personally identifiable real random numbers from both bioelectrical and physical signals.

## 1. Introduction

Random numbers are needed in some areas in computer science, such as authentication, secret key generation, game theory, and simulations. In these applications, particularly numbers should have good statistical properties and be unpredictable and nonreproducible. The number generation in the literature is performed in two different ways as deterministic and nondeterministic [1, 2]. PRNGs (Pseudo Random Number Generators), which are deterministic random number generators, generate numbers with fast, easy, inexpensive, and hardware independent solutions. The statistical qualities of these numbers produced are close to the ideal. PRNGs must meet the requirements specified in Table 1 to be used especially for authentication and key generation [3–5]. Therefore, nondeterministic functions are added to the output functions of PRNGs to guarantee these requirements.

TRNGs (True Random Number Generators), which are nondeterministic random number generators, present slower, more expensive, and hardware-dependent solutions compared to PRNGs. Contrary to PRNGs, there is no need to include extra components in the TRNG system designs for R2, R3, and R4 requirements. Because of the unpredictability of random numbers generated by the use of high noise sources with high entropy in TRNGs, it is assumed that the R2 requirement is met. If the R2 requirement is satisfied, then it is assumed that the R3 and R4 requirements are also satisfied. To meet the R1 requirement in TRNGs, postprocessing techniques are applied on the random numbers obtained by sampling from noise sources. This eliminates the statistical weaknesses of random numbers at the output of the TRNG. In addition, postprocessing techniques eliminate potential weaknesses and make TRNG designs strong and flexible [6, 7].

Recently, there have been studies performed on random number generation from human-based noise sources [8–12]. Elham et al. showed that two different people would produce different random numbers and that these numbers could be used as biometric signatures [8]. Xingyuan et al. proposed a TRNG structure using a one-dimensional chaotic map based on mouse movements. The proposed structure showed that NIST tests were successful and could be used on personal PCs [9]. Hu et al. performed real random number generation by observing mouse movements of computer users. The statistical properties of the binary number generators generated from mouse movements of three different users were examined by the NIST test suite. Three chaos-based approaches were proposed to eliminate similar motions generated by the same user. Successful results were also achieved with these approaches [10]. Rahimi et al. used two different ECG signals for the cryptographic key generation and suggested two different approaches. The security analyses of keys obtained by both approaches were tested with distinctiveness, randomness, temporal variance, and NIST and successful results were obtained [11]. In the study performed by Chen et al. [12], random number generation was done from ECG signals and the analysis was tested by NIST test suite. It was revealed by the authors that the PRNG-based generated numbers had more successful results in classical PRNG structures. Dang et al. showed the possibility of random number generation from EEG signals. Four different EEG datasets were used to illustrate the use of obtained numbers in cryptography applications and their statistical properties were analyzed with the NIST test suite. In this PRNG-based approach, the samples consisting of EEG signals were transformed into 0 and 1 number generators. Mathematical definitions of the structure using modular arithmetic for the transformation of number generators were given. In the study, it was shown that EEG signals could be used for random number generation. The NIST test suite was used for this purpose and a success of higher than 99% was achieved [13]. In a study carried out by Chen et al. [14], the authors showed that EEG signals agreed with Gaussian distribution and also revealed whether random number could be generated from signals. They used the EEG signals obtained from both healthy and sick people for the PRNG number generation. They used NIST test suite for statistical analysis and they failed some tests. It was shown as a result of the study that the generated numbers could be used as a PRN. In a study done by Chen et al. [15], random number generation was performed by using white noise signals taken from MPEG-1, WEBCAM, and IPCAM video files and it was emphasized that successful results were obtained from statistical tests. Buhanuponp et al. proposed a new encoding method for random number generation using EEG signals. This number generator, which can be used in low cost and real applications, is based on TRNG. A success of 99.47% was obtained from statistical tests. It was revealed that it was possible to do simple and fast bit generation by encoding method [16]. The summary of the literature methods used for random number generation is shown in Table 2.

In this article, it was shown that it was possible to generate real random numbers from personally identifiable bioelectrical signals (EEG, EMG, and EOG) and physical information (blood volume pulse, GSR (Galvanic Skin Response), and respiration). The accuracy of random numbers obtained was analyzed by NIST SP 800-22, scale index, and autocorrelation tests that are commonly used in the literature and the results are given in tables. The contributions made to the literature in this article can be summarized as follows:

1. It was shown that it was possible to generate personally identifiable random numbers.
2. Random numbers were generated with the TRNG structure.
3. It was revealed that random numbers can be generated by not only bioelectrical signals but also physical signals.
4. The analyses of statistical properties were performed and successful results were obtained. Analyses were also performed by scale index and autocorrelation tests in addition to the NIST test.

The article is organized as follows to achieve the aim.

In the second section, the structures and properties of PRNG and TRNG are briefly explained. Moreover, the comparisons of these two structures are presented in tabular form. In the third section, bioelectrical and physical signals are briefly described and the properties of signals and the dataset used in the study are given. In the fourth section, the proposed TRNG structure, the normalization for number generation, and sampling and postprocessing operations are presented. The tests used for the statistical analysis of the numbers and the results obtained are tabulated in Section 5. In the last section of the article, the results are discussed and the suggestions are made about future works.

## 2. Random Number Generation Methods

**Figure 1:**
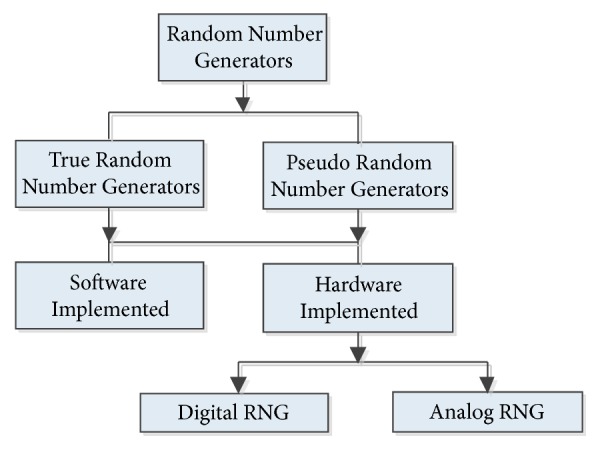
Random number generator classes.

Random numbers are widely used in areas such as cryptography and data transmission, luck games, secure communication, simulation, and game programming, where key generation is important. Random number generators can be divided into two classes: TRNG (True Random Number Generator) and PRNG (Pseudo Random Number Generator). Random numbers can be generated as hardware and software. The random numbers generated by the software can be defined by a specific mathematical model. On the other hand, it is possible to generate numbers by hardware with the help of noise source whose behavior cannot be predicted.  Figure 1 shows the classification of random number generation.

### 2.1. PRNG

**Figure 2:**
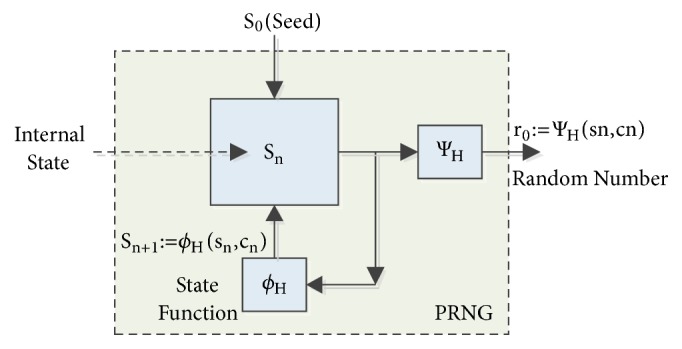
The general design architecture of PRNG.

The general design architecture of PRNG is shown in Figure 2. *r*_1_,*r*_2_,…  …  .  .  *r*_*n*_ ∈ *R* represents random number generator while *S*_*n*_ ∈ *S* indicates the internal states of pure PRNG and *P*_*s*_ is defined as the probability distribution of random seed. PRNG generates *r*_*n*_ random number from the current *S*_*n*_ state provided that Ψ∶*S* → *R* output function will be *r*_*n*_ = Ψ(*S*_*n*_). After that, using Φ transition function, *S*_*n*_ state is updated as *S*_*n*+1_= Φ (*S*_*n*_). *S*_0_ represents the first internal state and *S*_1_ value corresponds to the seed value of *S*_0_ state and the equation *S*_1_= Φ (*S*_0_) is generated [18]. In short, these generators need the starting parameters also known as seed. Random number generators with good quality statistics are generated by expanding these parameters with deterministic ways [19].

### 2.2. TRNG

**Figure 3:**
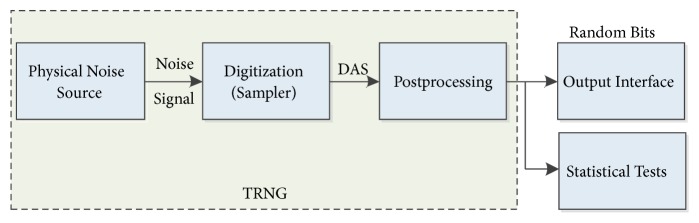
Block structure of TRNG.

The general design architecture of TRNG is shown in Figure 3. The values obtained by sampling noise sources are called digitalized analog signals (DAS). DAS random numbers correspond to a particular case of pure random numbers and they are subjected to algorithmic postprocessing to reduce their potential weaknesses. During this application, however, the output bit rate is reduced and the operating speed decreases.

The structural comparison of PRNG and TRNG number generators is shown in Table 3. According to Table 3, PRNGs generate fast, easily designable, and periodic numbers. On the other hand, TRNGs generate unpredictable, entropy dependent, and nonperiodic numbers. Beside these advantages, they are disadvantageous compared to PRNGs because they are hardware dependent and operate slowly.

## 3. Bioelectrical and Physical Signals

Bioelectrical signals are low amplitude noises between 100 *μ*V and 1 mV and are taken from the body through electrodes. The frequency spectra of such signals are in the low frequency range of 0.1 Hz ~ 2000 Hz. The amplitude and frequency characteristics of different bioelectrical signals taken from the body are shown in Table 4.

During brain activity, continuous rhythmic electrical potentials are produced and also electrical signals are generated due to receptor activity. The recording of these electrical signals with the electrodes embedded in the skull is called electroencephalography (EEG). The amplitudes of EEG waves range from 5 to 400 *μ*V and their frequencies change between 0.5 and 100 Hz. EEG signals are taken according to Extended International 10–20 system.

Electromyography (EMG) is a neurological examination method based on examining the electrical potentials of nerves and muscles. EMG is made in two ways by using surface electrodes and needle electrodes. In the tests using surface electrodes, electrodes are bonded to the skin surface. Superficial EMG can help monitor muscle and nerve disorders. In the tests using needle electrodes, needle electrodes are pricked into muscle tissue and electrical signals are recorded on muscle fibers. The amplitudes of EMG waves change between 100 *μ*V and 1 mV and their frequencies range from 10 to 500 Hz.

Electrooculogram (EOG) signals are corneal-retinal signals between the cornea and retina formed by eye movements and caused by hyperpolarization and depolarization. EOG signals are taken with the help of electrodes placed around eyes. The EOG signals are in the frequency band of about 0.1–10 Hz and their amplitudes are about 0.001–100 mV. Horizontal and vertical EOG signals vary with eye movement. One degree of movement causes a variation of 16 *μ*V in horizontal amplitude and 14 *μ*V in vertical amplitude.

Among physical signals, the blood volume pulse (BVP) is used to measure heart rate. BVP measurement is obtained using a photoplethysmography (PPG) sensor. This sensor measures changes in blood volume corresponding to changes in heart rate in arteries and capillaries and blood flow. The GSR signal is one of the most sensitive indicators of emotional stimulation to show whether individuals are under stress. It gives information about the conductivity of the skin. Another physical signal, respiration, is caused by the difference between breathing air and exhaling air. With the temperature converter, the heat exchange during respiration is converted into electrical activity.

**Figure 4:**
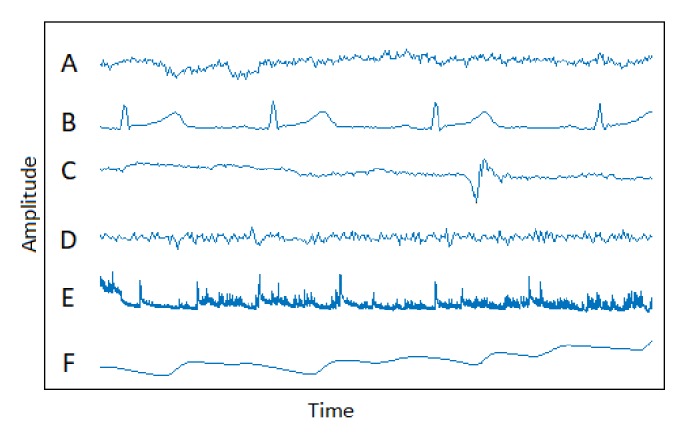
Bioelectrical and physical signals from database.

Figure 4 shows the samples bioelectrical and physical signals in the BNCIHORIZON2020 database. In this figure, the six rows of signals from top to bottom are samples EEG, EOG, EMG, GSR, and blood pressure respiration signals from the datasets A, B, C, D, E, and F, respectively.  Table 5 shows mean and standard deviation of bioelectrical and physical signals.

## 4. Personally Identifiable Number Generation from Bioelectrical and Physical Signals

In this study, to generate TRNG-based random numbers, bioelectrical and physical signals obtained from BNCIHORIZON2020 database were used. The overall number of the databases used is fifteen. These data are four EEG, four EMG, four EOG, one blood volume pulse, one GSR (Galvanic Skin Response), and one respiration. For EEG data, the signals in the database were recorded using thirty-two Ag/AgCl active electrodes with a sampling frequency of 512 Hz according to Extended International 10–20 system. EOG and EMG signals were recorded over the left and right flexor digitorum profundus. GSR, blood volume pulse, and respiration data were recorded simultaneously with bioelectrical signals.

**Figure 5:**
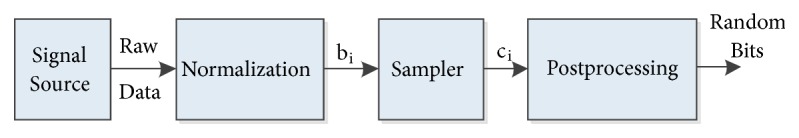
The steps of number generation.

Real random number generation from these data includes three steps, as shown in Figure 5. These steps are normalization, digitization (sampler), and postprocessing.

In normalization, all data obtained from noise sources (raw signals) first should be transformed into binary number system. To achieve this, the operations given with their mathematical explanations below are applied. Let each signal obtained from individuals be* x=(x*_*1*_*,x*_*2*_*,…x*_*n*_)^*T*^. Each sample is expressed as 5 bits by using modular arithmetic as shown in

(1) $$\begin{array}{c} y_{i}=x_{i}\mathrm{mod}32\;for\;\;i\in \left[1,n\right]. \end{array}$$

To produce 0 and 1 from 5-bit* y*_*i*_ generators,* z*_*i*_ generator is obtained according to (2). Each element of* z*_*i*_ generator is in a range of [0−5].

(2) $$\begin{array}{c} z_{i}=\overset{5}{\underset{k=1}{\sum }}y_{i_{k}}. \end{array}$$

Lastly,* b*_*i*_ random binary generator is obtained by using* z*_*i*_ generator with the help of (3). The algorithm of normalization is given below.

(3) $$\begin{array}{c} b_{i}=z_{i}\mathrm{mod}2\;for\;\;i\in \left[1,n\right]. \end{array}$$

After this step, sampling is applied to* b*_*i*_ generator obtained from bioelectrical and physical signals that are used as the noise source. Periodic and nonperiodic signals are used in the literature for sampling [17, 20]. In this article, logistic map presenting nonperiodic behavior was used for sampling. Logistic map presenting chaotic behavior is defined as

(4) $$\begin{array}{c} a_{i+1}=r*a_{i}*\left(\;1-a_{i}\;\right)\;i=0,1,2,\ldots \end{array}$$

where* r* is the system parameter,* a*_*1*_ is the seed or initial value, and* i* is the number of iterations. The numbers produced by logistic map and in a range of [0, 1] are real. Let these numbers be _a1_, _a2_, _a3_…. The expression “*a*_*i*_ >=0.5” produced at the i^th^ iteration indicates that the output of logistic map is assumed to be 1; otherwise it is 0. For* r*=3.91 and* a*_*1*_=0.2, first fifteen values are 0.2, 0.625, 0.916, 0.301, 0.823, 0.568, 0.959, 0.153, 0.508, 0.977, 0.087, 0.311, 0.837, 0.533, and 0.973. The produced sampling signal will be as follows: 0,1,1,0,1,1,1,0,1,1,0,0,1,1,1. When the sampling signal becomes 1, then* d*_*j*_=*b*_*i*_ is satisfied. When it is 0,* b*_*i*_ signal is neglected. This phenomenon is illustrated in Table 6. Sampling operation is given in Algorithm 2.

Postprocessing is applied to improve statistical properties of sampled random number generators. XOR, Von Neumann, LFSR, and Hash function structures are commonly used in the literature for postprocessing [21, 28]. In this study, XOR postprocessing was used. When sequential numbers are 0,1 and 1,0 with XOR in the random number generator, real random number is assumed to be 1 and otherwise 0. When XOR postprocessing is applied to the sampled 1110110010 number generator obtained in Table 5, the number generator obtained will be 01001.The algorithm for postprocessing is given in Algorithm 3.

## 5. Statistical Analysis of Random Numbers

NIST SP 800-22, scale index, and autocorrelation tests—commonly used in the literature—were used for the statistical analysis of generated random numbers.

### 5.1. NIST SP 800-22 Statistical Test Suite

NIST SP 800-22 is a test commonly used for statistical analysis of the numbers obtained from TRNG, PRNG, Physical Unclonable Function (PUF), and their hybrid generators [21–23]. NIST test suite includes fifteen tests and the parameters of each test are given in the related study [29]. The *α* value known as the level of significance is one of the most important parameters in the test. The selection of *α* as 0 indicates that the randomness of numbers to be tested has a confidence value of 99%. Another parameter is p value and it is known as the measure of randomness. If this value is equal to 1, numbers are said to have perfect randomness. If p value becomes 0, numbers are not random. The *α* value of personally identifiable random numbers to be used for key and verification applications should be appropriately selected. For each test, if p value is greater than or equal to *α* value, then the test is successful. Otherwise the test becomes unsuccessful; i.e., the numbers generated are not random. Generally, *α* is selected from [0.001, 0.01] range.

NIST test suite analysis results of personally identifiable random numbers obtained from bioelectrical and physical signals in the dataset are shown in Tables 7 and 8, respectively. As can be seen from tables, all data used was successful in the NIST test because their p value was higher than 0.01.

### 5.2. Scale Index Test

The scale index test was applied for statistical analysis of numbers. The scale index technique was proposed by Benitez [30]. This technique was used to determine the information about the degree of nonperiodicity of a signal or generated number series. In literature, for determining the periodicity of TRNG and PRNG, the scale index test was used [24, 25]. The scale index was based on the continuous wavelet transform and wavelet multiresolution analyses. The scales* s* and* f* at time* u* in the continuous wavelet transform (CWT) and scalogram were shown as given in equations (5) and (6) [22]. One has

(5) Wfu,s≔f,ψu,s=∫−∞+∞ftψu,s∗tdt

(6) s≔Wfu,s=∫−∞+∞Wfu,s2du

The continuous wavelet transform's energy of* f* at a scale* s* was illustrated as* S(s)*. Equation (7) shows the inner scalogram of* f* at a scale* s*.

(7) $$\begin{array}{c} S^{in}\left(\;s\;\right)≔\left.\;{\left\|\;Wf\left(\;u,s\;\right)\;\right\|}_{j\left(s\right)}={\left(\;\int_{c\left(s\right)}^{d\left(s\right)}{\left|\;Wf\left(\;u,s\;\right)\;\right|}^{2}du\;\right)}^{2}\;\right. \end{array}$$

where *J*(*s*) = [*c*(*s*), *d*(*s*)]⊆*I* is the maximal subinterval in* I* for which the support of *ψ*_*u*,*s*_ is included in* I* for all* u ϵ j(s).* Considering that the length of* J (s)* depends on the scale* s*, the values of the inner scalogram at different scales cannot be compared. The normalized* s*^*in*^ is defined as shown in

(8) $$\begin{array}{c} {\overset{-}{S}}^{in}\left(\;s\;\right)=\frac{S^{in}\left(s\right)}{{\left(d\left(s\right)-c\left(s\right)\right)}^{1/2}}. \end{array}$$

Equation (9) shows the scale index of* f* in the scale interval [s_0_, s_1_].

(9) $$\begin{array}{c} i_{scale}≔\frac{S\left(s_{min}\right)}{S\left(s_{max}\right)}. \end{array}$$

The degree of nonperiodicity of bioelectrical and physical magnitudes was determined by the scale index test whose details were explained above.  Table 9 shows obtained scale index values.

The scale index value* i*_*scale*_ should be in the range of* 0≤ i*_*scale*_*≤1.* If the scale value obtained from the generated system is 0 or near 0, then the system is defined as periodic and if 1 or near to 1, then it is defined as nonperiodic. According to Table 8, it was observed that the results obtained from both bioelectrical and physical signals were successful in scale index test and they were close to 1.

### 5.3. Autocorrelation Test

Finally, to observe the variations of 0 and 1 s in the generated random numbers the autocorrelation test was used [26, 27].

Equation (10) shows the mathematical definitions of the test [31].

(10) $$\begin{array}{c} A\left(\;d\;\right)=\overset{n-d-1}{\underset{i=0}{\sum }}b_{i}⊕b_{i+d} \end{array}$$

where ⊕ is the XOR operator,* n *is the length of the generated number sequences, and* b*_*i*_ represents the number sequence. The* d *value is the constant integer and between [1,*(n/*2)]. Equation (11) shows the relationship between 0 and 1s.

(11) $$\begin{array}{c} X5=\frac{2\left[A\left(d\right)-\left(n-d\right)/2\right]}{\sqrt{n-d}}. \end{array}$$

For *α* = 0.05, if |X5|* <* 1.6449, then the test is successful.

Autocorrelation test was used to determine 0-1 change in the random numbers obtained from both bioelectrical and physical magnitudes.  Table 10 shows autocorrelation test results. As seen in Table 9, for each dataset, |X5| value is in the specified interval. Thus, random numbers obtained from both bioelectrical and physical signals were successful in autocorrelation test.

## 6. Discussion and Conclusion

Random numbers have been generated in the literature from bioelectrical signals such as EEG. These numbers have the PRNG structure. However, the statistical properties of the numbers generated from EEG signals are not good. In this article, the TRNG structure using bioelectrical and physical signals as a source of randomness was proposed. Although the taken signals are periodic, the level of noise that would emerge from any source on the signal causes the signal to be nonperiodic. In this case, the random numbers to be generated are unpredictable. Personally identifiable random numbers were generated from the obtained raw signals using normalization (see Algorithm 1), sampling, and postprocessing operations. The process is faster than the TRNG structures in the literature because of the simple structure of normalization, sampling, and postprocessing. NIST SP 800-22 test was used to show that the statistical properties of the generated numbers were improved; scale index test was used to reveal the level of nonperiodicity and autocorrelation tests were used to observe 0 and 1 change of numbers. All test results were presented in tabular form and all results were found to be successful. The results indicate that TRN generation from bioelectrical and physical signals obtained from the human body is possible.

Thus, the obtained random numbers are suitable for use in different areas, such as key generation, authentication, games of chance, simulation, and game programming. It is possible to carry out various studies on random number generation using these bioelectrical and physical signals as well as different types of signals like EGG and ECG. The present study will be the basis for random number generation using such signals.

## Figures and Tables

**Algorithm 1:**
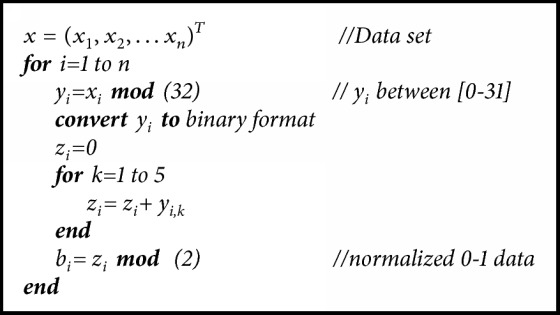
Normalization procedure.

**Algorithm 2:**
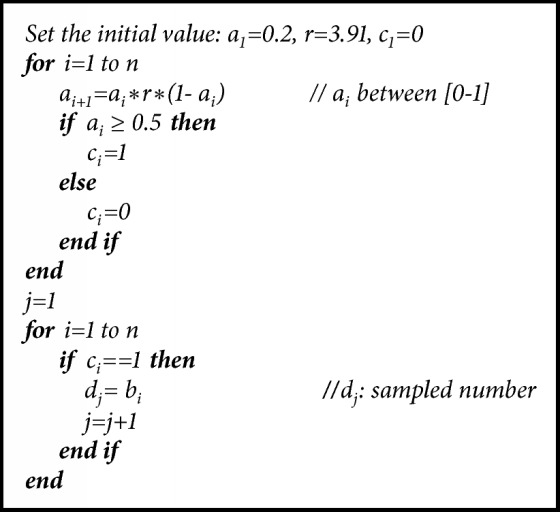
Sampler procedure.

**Algorithm 3:**
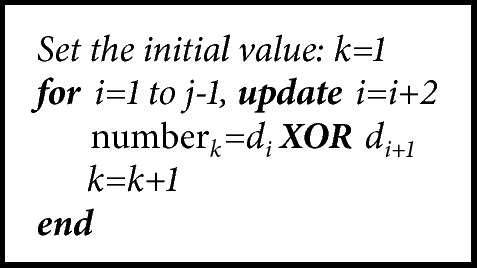
Postprocessing procedure.

**Table 1:** Requirements for random numbers.

**Requirement**	**Explanation**
**R1**	RNGs must generate random numbers having good statistical properties at the output to be used in cryptographic applications.

**R2**	In case of the attacker knows the sub-generators of random numbers, it must not be allowed to calculate or predict premise and consecutive random numbers with high accuracy.

**R3**	It must not be possible to predict or calculate previously generated random numbers with high accuracy by considering the known current internal state value of a RNG or without requiring its internal state information.

**R4**	It must not be possible to predict or calculate subsequent random numbers with high accuracy by considering the known current internal state value of a RNG or without requiring its internal state information.

**Table 2:** Properties of random generation methods.

References	Implementation type	Noise Source	Tests	Performance
[2]	PRNG	MARC-bb Algorithm	TestU01	success
[3]	TRNG	jitter	NIST, scale index, Autocorrelation	success
[4]	PRNG	Chaos	NIST	success
[5, 17]	TRNG	jitter	NIST	success (for some test)
[7]	PRNG&TRNG	Chaos and jitter	NIST	success
[9]	TRNG	Chaotic signal	NIST	success
[10]	TRNG	Mouse movement	NIST	success
[11, 12]	PRNG	ECG	NIST	success
[13]	PRNG	EEG	NIST	success
[14]	PRNG	EEG	NIST	success (for some test)
[15]	TRNG (AVRNG)	Audio and Video	NIST	success
[16]	TRNG	EEG	NIST	success
[18]	TRNG&PRNG	Chaotic signal	NIST, FIPS	success
[19]	PRNG	Boolean function	NIST	success (for some test)
[20]	TRNG	Chaotic signal	NIST,TESTU01, scale index	success
[21, 22]	TRNG	jitter	NIST	success
[23]	PRNG	jitter	NIST	success
[24]	PRNG	chaotic maps	NIST, scale index	success
[25]	TRNG	chaotic maps	NIST, scale index	success
[26]	TRNG	Intel DRNG	TestU01, Autocorrelation	success
[27]	TRNG	Telegraph Noise	NIST, Autocorrelation	success

**Table 3:** Comparison ofTRNG and PRNG.

	**TRNG**	**PRNG**
Realization type	Hardware required	Optional
Periodicity	Aperiodic	Periodic
Ease of application of design cycle	Complex	Easy because of standard structures
Efficiency	Weak	Perfect
Change of theoretical calculation limit	Constant (independent of time)	Dependent on time
Cryptographic security requirement	R1, R2 requirement	R1, R2, R3, and R4 requirements

**Table 4:** Bioelectrical signals and their properties.

**Signal Type**	**Amplitude**	**Frequency**
EEG Electroencephalogram	2-100 *μ*V	0.5-50 Hz
EMG Electromyogram	100 *μ*V-1 mV	10- 500Hz
EOG Electrooculography	0.001-100 mV	0.1-10 Hz

**Table 5:** Bioelectrical signals and their properties.

**Features **	**EEG**	**EMG**	**EOG**	**GSR**	**Blood Pressure**	**Respiration**
Mean	-1.26x10^4^	-1.825 x10^4^	3.104	150.11	-1.085 x10^5^	-1.397 x10^5^
Standard deviation	6.98 x10^3^	6.862 x10^3^	10.652	1.999	3.75 x10^3^	1.544 x10^4^

**Table 6:** Obtaining the sampled signal.

**Signals**	**Obtained Bits**
b_i_ (Normalized signals)	0,1,1,1,1,0,1,0,1,0,0,1,0,1,0
c_i_ (Sampled signal obtained from Logistic map)	0,1,1,0,1,1,1,0,1,1,0,0,1,1,1
d_j_ (Sampled signal)	1,1, 1,0,1 1,0 0,1,0

**Table 7:** NIST test results obtained from bioelectrical signals.

**Test Type**	**EEG1**	**EEG2**	**EEG3**	**EEG4**	**EMG1**	**EMG2**	**EMG3**	**EMG4**	**EOG1**	**EOG2**	**EOG3**	**EOG4**
Frequency Monobit	*0.4564*	0.016	0.539	0.784	0.324	0.523	0.663	0.961	0.701	0.406	0.047	0.737
Frequency Test within a Block	0.492	0.184	0.252	0.744	0.253	0.594	0.294	0.058	0.805	0.916	0.725	0.264
Runs	0.445	0.221	0.358	0.734	0.676	0.453	0.506	0.258	0.440	0.246	0.842	0.388
Longest Run of Ones in a Block	0.277	0.428	0.295	0.131	0.853	0.046	0.094	0.221	0.087	0.871	0.616	0.973
Binary Matrix Rank	0.774	0.648	0.980	0.458	0.579	0.402	0.795	0.354	0.481	0.481	0.636	0.448
Discrete Fourier Transform	0.212	0.295	0.798	0.784	0.996	0.964	0.408	0.108	0.032	0.773	0.531	0.348
Non Overlapping Template Matching	*0.234*	0.451	0.553	0.947	0.880	0.925	0.218	0.431	0.477	0.397	0.568	0.843
Overlapping Template Matching	0.510	0.310	0.712	0.119	0.631	0.615	0.348	0.028	0.633	0.838	0.363	0.715
Universal	0.436	0.235	0.533	0.661	0.748	0.117	0.659	0.527	0.219	0.371	0.567	0.512
Linear Complexity	0.745	0.874	0.236	0.225	0.318	0.374	0.453	0.178	0.959	0.985	0.406	0.210
Serial	0.560	0.026	0.540	0.909	0.565	0.616	0.723	0.526	0.714	0338	0.138	0.651
	/0.437	/0.211	/0.357	/0.734	/0.679	/0454	/0.498	/0.257	/0.468	/0.224	/0.826	/0.388
Approximate Entropy	0.254	0.330	0.690	0.532	0.840	0.305	0.026	0.757	0.345	0.324	0.176	0.710
Cumulative Sums	0.469	0.014	0.171	0.875	0.503	0.802	0.410	0.405	0.894	0.416	0.042	0736
Random excursions test^*∗*^	0.712	0.337	0.501	0.287	0.746	0.253	0.413	0.682	0.485	0.226	0.711	0.603
Random excursions variant test^*∗*^	0.519	0.483	0.645	0.831	0.452	0.846	0.445	0.577	0.186	0.705	0.694	0.279

^*∗*^More than one p value was obtained and it was found that p value > 0.01. The values given for these tests are average.

**Table 8:** NIST test results obtained from physical signals.

**Test Type**	**GSR**	**Blood Pressure**	**Respiration**
Frequency Monobit	0.715	0.079	0.951
Frequency Test within a Block	0.125	0.213	0.412
Runs	0.445	0.490	0.290
Longest Run of Ones in a Block	0.230	0.762	0.639
Binary Matrix Rank	0.490	0.232	0.208
Discrete Fourier Transform	0.916	0.469	0.826
Non Overlapping Template Matching	0.817	0.734	0.140
Overlapping Template Matching	0.415	0.040	0.962
Universal	0.043	0.115	0.636
Linear Complexity	0.388	0.560	0.680
Serial	0.698	0.166	0.578
	/0.444	/0.473	/0.295
Approximate Entropy	0.422	0.0.485	0.092
Cumulative Sums	0.773	0.055	0.345
Random excursions test^*∗*^	0.252	0.289	0.601
Random excursions variant test^*∗*^	0.333	0.427	0.397

^*∗*^More than one p value was obtained and it was found that p value > 0.01. The values given for these tests are average.

**Table 9:** Scale index test results of datasets.

	**1. Dataset**	**2. Dataset**	**3. Dataset**	**4. Dataset**
EEG	0.924	0.936	0.973	0.951
EMG	0.944	0.955	0.957	0.924
EOG	0.756	0.763	0.861	0.804
Blood Pressure	0.936			
GSR	0.964			
Respiration	0.879			

**Table 10:** Autocorrelation test results of datasets.

**d**		**8**	**10**	**13**
EEG datasets	1	0.205	0.445	0.302
	2	1.289	0.159	0.896
	3	0.216	0.285	1.169
	4	0.627	0.776	0.599

EMG datasets	1	1.175	0.593	0.633
	2	0.253	1.08	0.382
	3	1.261	0.688	0.991
	4	0.836	1.498	1.137

EOG datasets	1	1.068	0.812	0.107
	2	1.089	1.431	0.727
	3	1.497	0.927	1.182
	4	0.896	0.346	0.111

Blood Pres.	1	1.381	0.468	0.359

GSR	1	0.833	0.890	0.188

Respiration	1	0.730	0.993	1.030

## Data Availability

The data used to support the findings of this study are available from the corresponding author upon request.
